# Association between red blood cell distribution width-to-albumin ratio and prognosis of patients with acute myocardial infarction

**DOI:** 10.1186/s12872-023-03094-1

**Published:** 2023-02-03

**Authors:** Hongwu Li, Yinjun Xu

**Affiliations:** 1 Department of Cardiology, Peking Union Medical College Hospital, Beijing, 100730 People’s Republic of China; 2 Department of General Practice, Lin’an People’s Hospital Affiliated to Hangzhou Medical College, The First People’s Hospital of Lin’an District, No.548 Yijin Street, Lin’an District, Hangzhou, 311300 Zhejiang Province People’s Republic of China

**Keywords:** The red blood cell distribution width-to-albumin ratio, Acute myocardial infarction, 90-day mortality, MIMIC-III, Relationship

## Abstract

**Background:**

Red blood cell distribution width (RDW) and albumin level were considered to be related to the prognosis of patients with acute myocardial infarction (AMI). This study aims to investigate the correlation between RAR and 90-day mortality in AMI patients.

**Methods:**

Data of AMI patients were obtained from the Medical Information Mart for Intensive Care III (MIMIC-III) database. According to the median, RAR < 4.32 was regarded as low RAR level group, and RAR ≥ 4.32 as high RAR level group; low RDW level group was defined as < 14.00%, and high RDW level group as ≥ 14.00%; albumin < 3.30 g/dL was low level group, and albumin ≥ 3.30 g/dL as high level group. The outcome was the mortality rate within 90 days after admission to ICU. Univariate and multivariate Cox models were performed to determine the relationship between RAR and 90-day mortality in AMI patients with hazard ratio (HR) and 95% confidence interval (CI). Stratification analyses were conducted to explore the effect of RAR on 90-day mortality in different subgroups of age, gender, simplified acute physiology score II (SAPS II), elixhauser comorbidity index (ECI) score, treatment modalities and white blood cell.

**Results:**

Of the total 2081 AMI patients, 543 (26.09%) died within 90-day follow-up duration. The results showed that high RAR (HR = 1.65, 95% CI 1.34–2.03) and high RDW levels (HR = 1.31, 95% CI 1.08–1.61) were associated with an increased risk of death in AMI patients, and that high albumin level was related to a decreased risk of death (HR = 0.77, 95%CI 0.64–0.93). The relationship of RAR level and the mortality of AMI patients was also observed in the subgroup analysis. Additionally, the finding indicated that RAR might be a more effective biomarker for predicting 90-day mortality of AMI patients than albumin, RDW.

**Conclusion:**

RAR may be a potential marker for the prognostic assessment of AMI, and a high RAR level was correlated with increased risk of 90-day mortality of AMI patients.

## Background

Acute myocardial infarction (AMI) is a type of myocardial necrosis caused by acute coronary artery occlusion [1], which is the leading cause of hospitalization and death in the world [2]. Despite advances in some treatments, the mortality rate from AMI still remains high, with a 90-day mortality rate of 12.2% [3, 4]. Timely identification of influencing factors associated with death in patients with AMI is of great significance to improve prognosis and provide better guidance for treatment.

Red blood cell distribution width (RDW) is considered as an indicator of the heterogeneity of red blood cell (RBC) volume in peripheral blood [5, 6]. Existing evidence suggests that RDW has been a novel biomarker of inflammation and oxidative stress [6, 7]. Previous studies have reported that higher RDW value was independent risk factor for poor prognosis in many diseases, such as AMI [5], atrial fibrillation [8], ischaemic stroke [9]. Recently, some recommendations have indicated that combining RDW with some biomarkers may be more valuable for disease prognosis. Albumin, an important protein that can reflect the nutritional and inflammatory status [10, 11], has been shown to be associated with anti-inflammatory activity [12] and reduction of oxidative stress [13]. A cohort study in 2,305 patients with first-onset AMI showed that low serum albumin levels at admission was related to an increased risk of long-term all-cause, cardiovascular, and cardiac mortality in patients [14]. Several studies have examined the relationship between prognosis of circulation system diseases and the combined marker-RDW/albumin ratio (RAR). In the study of Zhao et al., they concluded that a high RAR level was associated with an increased mortality among intensive care unit (ICU) patients with stroke [15]. Furthermore, an elevated RAR level was also regarded as an independent risk factor of all-cause mortality in patients with thoracic or abdominal aortic aneurysm [16]. However, to our knowledge, there are few relevant studies investigating the association between RAR and the prognosis of AMI patients to date.

Herein, the aim of this study was to explore the correlation between RAR and 90-day mortality in patients with AMI.

## Methods

### Study population

This cohort study collected the information of participants from the Medical Information Mart for Intensive Care III (MIMIC-III) database 2001–2012. This large, single-center database records the medical information over forty thousand patients admitted to ICU between 2001 and 2012 [17], which includes some information, such as demographic data, vital signs, laboratory test, and survival data.

International Classification of Diseases-9 (ICD-9) diagnosis codes (41000–41092) were used to identify patients with AMI in the MIMIC-III database. The inclusion criteria were as follows: (1) patients diagnosed with AMI at ICU admission; (2) patients aged ≥ 18 years old at diagnosis; (3) patients with measurement of RDW and albumin. The exclusion criteria were as follows: (1) length of ICU stay < 24 h; (2) patient’s individual data with missing. This study was conducted in accordance with the Declaration of Helsinki. The MIMIC-III database was approved by the Institutional Review Boards of the Massachusetts Institute of Technology and Beth Israel Deaconess Medical Center. https://mimic.mit.edu/docs/iii/. Informed consent has been obtained from all participants.

### Data collection

The data of patients were extracted from the MIMIC-III database: (1) demographics: age, gender, race and marital status; (2) vital signs: respiratory rate (times/min), heart rate (times/min), systolic blood pressure (SBP, mmHg), diastolic blood pressure (DBP, mmHg) and pulse oximetry-derived oxygen saturation (SpO_2_, %); (3) laboratory parameters: sodium (mEq/L), potassium (mEq/L), calcium (mEq/L), creatine kinase isoenzymes (CK-MB, ng/mL), pH, white blood cell (WBC, K/uL), glucose (mg/dL), creatinine (mg/dL), blood urea nitrogen (BUN, mg/dL), bicarbonate (mEq/L), hemoglobin (g/dL), albumin (g/dL), RDW (%); (4) scoring systems: sequential organ failure assessment (SOFA), simplified acute physiology score II (SAPS II), glasgow coma scale (GCS), elixhauser comorbidity index (ECI). (5) Treatment modalities: percutaneous coronary intervention (PCI), coronary artery bypass graft (CABG), and thrombolysis. (6) Vasopressor use and ICU length (days). Vital signs and laboratory parameters recorded within 24 h of initial admission were used for analysis.

### Assessment of RAR

RAR was defined as the ratio of the RDW (%) to albumin (g/dL). We divided RAR levels into two groups based on the median: RAR < 4.32 was regarded as low RAR level group, and RAR ≥ 4.32 was regarded as high RAR level group. Similarly, albumin levels were divided into low albumin level group (albumin < 3.30 g/dL) and high albumin level group (≥ 3.30 g/dL) based on median. Low RDW level group was defined as < 14.00%, and high RDW level group as ≥ 14.00%.

### Outcome and follow-up

The endpoint event of this study was the mortality of AMI patients within 90 days after admission to ICU. The maximum follow-up duration was 90 days, and the median follow-up time was 90 (75, 90) days.

### Statistical analysis

Measurement data with normal distribution was described by the mean ± standard deviation (Mean ± SD), and measurement data with non-normal distribution by the median and quartile spacing [M (Q1, Q3)]. The categorical data was depicted by the number of cases and composition ratio n (%). Baseline characteristics of participants were compared between survival group and death group by using either Mann–Whitney U rank-sum test or χ^2^ test.

Univariate and multivariate Cox models were used for assessing the relationship between RAR and 90-day mortality in patients with AMI, and hazard ratio (HR) with 95% confidence interval (CI) was calculated. Model 1 was the univariate Cox model (unadjusted); covariates in model 2 were adjusted for age, vasopressor use, SOFA score, SAPS II score, GCS score and ECI score; covariates in model 3 were adjusted for age, marital status, respiratory rate, heart rate, DBP, WBC, potassium, calcium, pH, creatinine, bicarbonate, hemoglobin, SpO_2_, vasopressor use, SOFA score, SAPS II score, GCS score ECI score, PCI, CABG and ICU length. Stratification analyses were conducted to explore the effect of RAR between different subgroups (age, gender, SAPS II score, ECI score, treatment modalities and WBC). Box plots and t-test were constructed to observe the positive correlation between RAR and ECI score, SAPS II score and SOFA score. We adopted Kaplan–Meier survival curve to analyze survival status in both the low and high RDW level groups, both the low and high albumin level groups, and both the low and high RAR level groups. A restricted cubic spline (RCS) model was used to observe whether there was a linear relationship between RAR and 90-day mortality of patients with AMI. Lastly, the total samples were randomly split into training set and testing set with the ratio of 7:3. A prediction model by using the RAR was established in the training set for the prediction of 90-day mortality of patients with AMI. The C-index was adopted to compare the predicting performance between developed RAR model and RDW-model, albumin-model in the testing set. All statistical analyses were conducted by using Rstudio (version 4.0.3) and SAS (version 9.4). *P* < 0.05 was considered statistically significant.

## Results

### Baseline characteristics

We excluded some patients who did not measure RDW and albumin (n = 1731) or whose baseline data were incomplete (n = 909) or whose ICU stay was less than 24 h (n = 244) or who were lost to follow-up (n = 52), 2081 AMI patients were included in the final analysis. They were divided into survival group (n = 1538) and death group (n = 543). The 90-day mortality of patients with AMI was 26.09%. As shown in Table 1, baseline characteristics of participants were compared between survival group and death group. We found that there were more AMI patients with high levels of albumin, low levels of RDW and RAR in the survival group than in the death group.

**Table 1:** Baseline characteristics of the study population

Characteristics	Total (n = 2081)	Survival group (n = 1538)	Death group (n = 543)	*P*
Gender, n (%)				0.108
Female	823 (39.5%)	592 (38.5%)	231 (42.5%)	
Male	1258 (60.5%)	946 (61.5%)	312 (57.5%)	
Age, years, n (%)				< 0.001
< 65	631 (30.3%)	559 (36.3%)	72 (13.3%)	
≥ 65	1450 (69.7%)	979 (63.7%)	471 (86.7%)	
Race, n (%)				0.207
Asian	34 (1.63%)	25 (1.63%)	9 (1.66%)	
Black	114 (5.48%)	89 (5.79%)	25 (4.60%)	
Other^#^	422 (20.3%)	296 (19.2%)	126 (23.2%)	
White	1511 (72.6%)	1128 (73.3%)	383 (70.5%)	
Marital status, n (%)				0.035
Married	1157 (55.6%)	867 (56.4%)	290 (53.4%)	
Other*	924 (44.4%)	671 (43.6%)	253 (46.6%)	
Respiratory rate, times/min, Mean ± SD	19.0 ± 6.80	18.3 ± 6.45	21.2 ± 7.29	< 0.001
Heart rate, times/min, Mean ± SD	88.3 ± 19.3	87.3 ± 18.6	91.1 ± 20.9	< 0.001
SBP, mmHg, Mean ± SD	122 ± 25.8	122 ± 25.0	120 ± 27.9	0.052
DBP, mmHg, Mean ± SD	62.8 ± 17.1	63.5 ± 16.5	60.8 ± 18.6	0.003
SPO_2_, %, Mean ± SD	96.7 ± 6.96	97.1 ± 6.09	95.7 ± 8.90	0.001
Sodium, mEq/L, Mean ± SD	138 ± 4.79	138 ± 4.42	138 ± 5.70	0.907
Potassium, mEq/L, Mean ± SD	4.36 ± 0.84	4.30 ± 0.81	4.53 ± 0.90	< 0.001
Calcium, g/dL, Mean ± SD	8.54 ± 0.97	8.58 ± 0.95	8.43 ± 0.99	0.002
CK-MB, ng/mL, Mean ± SD	77.1 ± 152	82.5 ± 147	63.6 ± 165	0.058
pH, Mean ± SD	7.36 ± 0.11	7.36 ± 0.10	7.34 ± 0.12	< 0.001
WBC, K/uL, Mean ± SD	12.6 ± 8.08	12.2 ± 7.49	13.9 ± 9.44	< 0.001
Glucose, mg/dL, Mean ± SD	179 ± 116	181 ± 120	175 ± 104	0.322
Creatinine, mg/dL, Mean ± SD	1.80 ± 1.96	1.70 ± 2.02	2.09 ± 1.74	< 0.001
BUN, mg/dL, Mean ± SD	33.0 ± 23.9	30.1 ± 21.9	41.3 ± 27.0	< 0.001
Bicarbonate, mEq/L, Mean ± SD	23.2 ± 4.94	23.4 ± 4.76	22.6 ± 5.39	0.005
Hemoglobin, g/dL, Mean ± SD	11.9 ± 2.16	12.0 ± 2.19	11.3 ± 1.96	< 0.001
Albumin, g/dL, n (%)				< 0.001
Low level	930 (44.7%)	599 (38.9%)	331 (61.0%)	
High level	1151 (55.3%)	939 (61.1%)	212 (39.0%)	
RDW, n (%)				< 0.001
Low level	1013 (48.7%)	831 (54.0%)	182 (33.5%)	
High level	1068 (51.3%)	707 (46.0%)	361 (66.5%)	
RAR, n (%)				< 0.001
Low level	1041 (50.0%)	885 (57.5%)	156 (28.7%)	
High level	1040 (50.0%)	653 (42.5%)	387 (71.3%)	
SAPS II, Mean ± SD	41.9 ± 13.8	39.1 ± 12.7	49.6 ± 13.7	< 0.001
SOFA total score, Mean ± SD	6.06 ± 3.47	5.63 ± 3.32	7.29 ± 3.60	< 0.001
GCS score, Mean ± SD	13.7 ± 2.81	13.9 ± 2.66	13.3 ± 3.17	< 0.001
ECI score, Mean ± SD	9.84 ± 8.02	8.55 ± 7.55	13.5 ± 8.18	< 0.001
PCI, n (%)				0.006
No	1567 (75.3%)	1134 (73.7%)	433 (79.7%)	
Yes	514 (24.7%)	404 (26.3%)	110 (20.3%)	
CABG, n (%)				< 0.001
No	1510 (72.6%)	1083 (70.4%)	427 (78.6%)	
Yes	571 (27.4%)	455 (29.6%)	116 (21.4%)	
Thrombolysis, n (%)				0.370
No	1961 (94.2%)	1454 (94.5%)	507 (93.4%)	
Yes	120 (5.77%)	84 (5.46%)	36 (6.63%)	
Vasopressor use, n (%)				< 0.001
No	1901 (91.4%)	1453 (94.5%)	448 (82.5%)	
Yes	180 (8.65%)	85 (5.53%)	95 (17.5%)	
ICU length, days, Mean ± SD	6.64 ± 7.40	5.97 ± 6.91	8.55 ± 8.35	< 0.001

SBP, systolic blood pressure; DBP, diastolic blood pressure; SpO2, pulse oximetry-derived oxygen saturation; CK-MB, creatine kinase isoenzymes; WBC, white blood cell; BUN, blood urea nitrogen; RDW, red blood cell distribution width; RAR, the ratio of the RDW to albumin; SAPS, simplified acute physiology score; SOFA, sequential organ failure assessment; GCS, glasgow coma scale; ECI, elixhauser comorbidity index; PCI, percutaneous coronary intervention; CABG, coronary artery bypass graft; ICU, intensive care unit. Other^#^ include Hispanic or Latino, American Indian/Alaska, unknown and multirace ethnicity; Other* include divorce, separate, single and widowed

### Relationship between RAR and 90-day mortality of patients with AMI

**Fig. 1:**
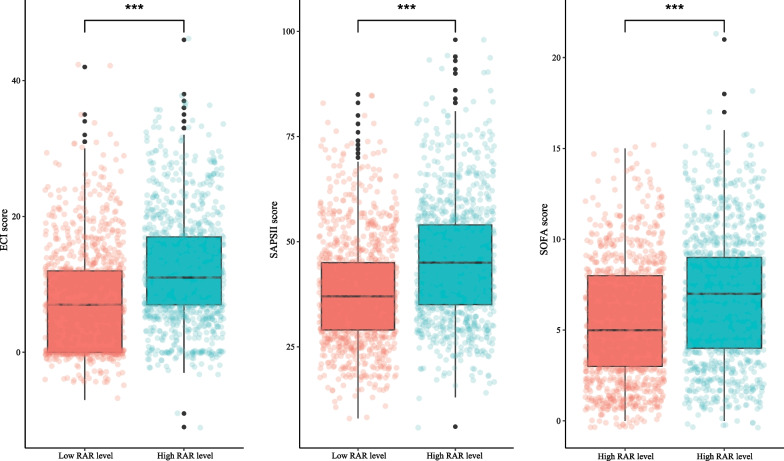
Box plots was constructed to observe the correlation between RAR and ECI score, SAPS II score and SOFA score, *** represents* P* < 0.001. RDW, red blood cell distribution width; RAR, the ratio of the RDW to albumin; ECI, elixhauser comorbidity index; SAPS, simplified acute physiology score; SOFA, sequential organ failure assessment

**Fig. 2:**
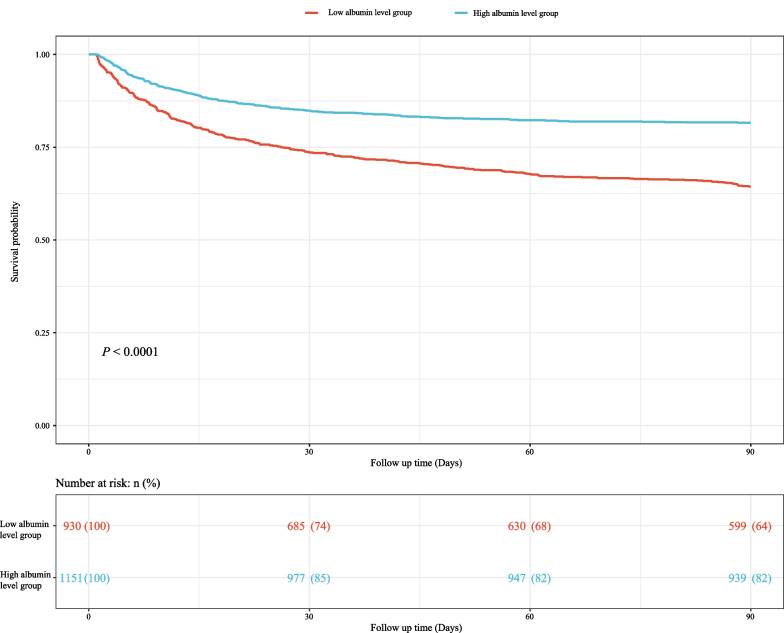

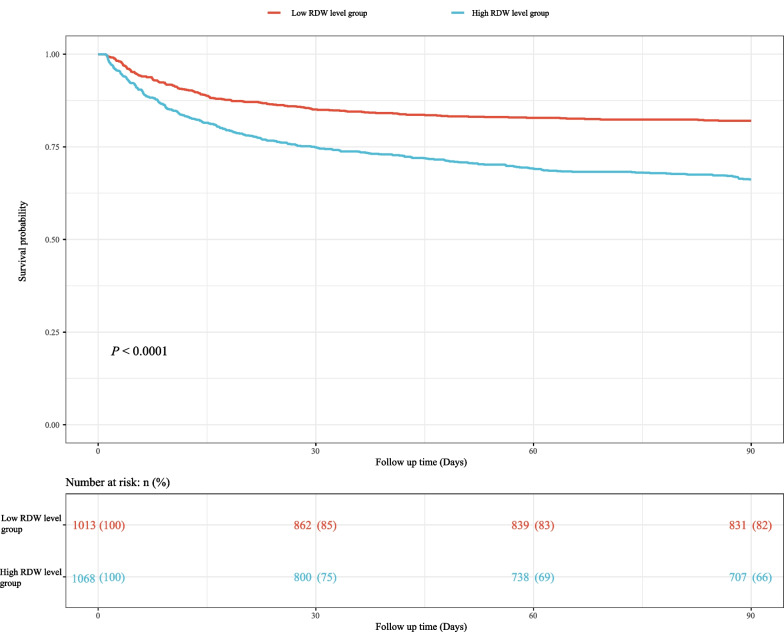

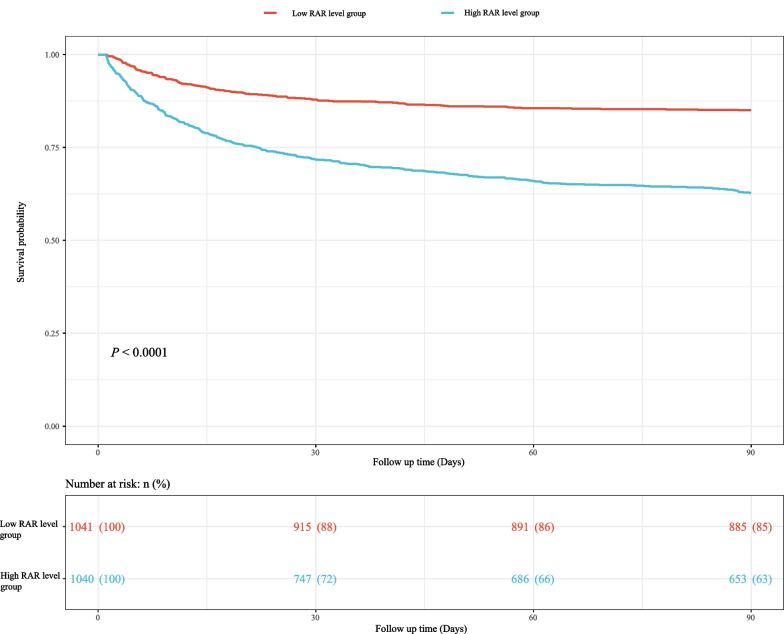
The survival curves of **a** both the low and high albumin level groups, **b** both the low and high RDW level groups and **c** both the low and high RAR level groups. RDW, red blood cell distribution width; RAR, the ratio of the RDW to albumin

**Fig. 3:**
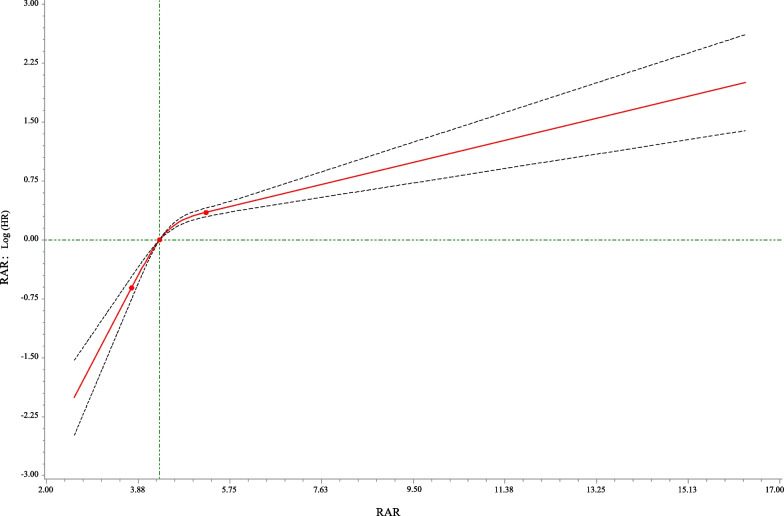
Association between the RAR and 90-day mortality of patients with AMI based on the restricted cubic spline model. RAR, the ratio of the RDW to albumin; AMI, acute myocardial infarction

We used Cox models to determine the association between RAR and 90-day mortality of patients with AMI. As illustrated in Table 2, the results suggested that the risk of death in AMI patients with high RAR level group was higher than that in patients with low RAR level group (Model 1: HR = 2.84, 95% CI 2.36–3.42, *P* < 0.001; Model 2: HR = 1.76, 95% CI 1.45–2.13, *P* < 0.001; Model 3: HR = 1.65, 95% CI 1.34–2.03, *P* < 0.001). Simultaneously, the relationship of RDW, albumin and 90-day mortality of patients with AMI were also investigated in the Table 2. These results indicated that high RDW level (Model 3: HR = 1.31, 95% CI 1.08–1.61, *P* = 0.007) was associated with an increased risk of death in AMI patients, and that high albumin level was related to a decreased risk of death (Model 3: HR = 0.77, 95% CI 0.64–0.93, *P* = 0.007). Figure 1 shows that RAR level was positively correlated with ECI score, SAPS II score and SOFA score, which suggested that RAR might was related to 90-day mortality of patients with AMI. Additionally, Fig. 2 presents the survival curves of both the low and high RDW level groups, both the low and high albumin level groups, and both the low and high RAR level groups, and the result indicated that the higher the RDW level or the lower the albumin level or the higher the RAR level, the worse the survival probability of AMI patients. The RCS model showed that the relationship between RAR and 90-day mortality of patients with AMI was nonlinear (Fig. 3).

**Table 2:** The association between albumin, RDW, RAR and 90-day mortality of patients with AMI by univariate and multivariate Cox models

Variables	Model 1		Model 2		Model 3	
	HR (95% CI)	*P*	HR (95% CI)	*P*	HR (95% CI)	*P*
Albumin						
Low level	Ref		Ref		Ref	
High level	0.47 (0.40–0.56)	< 0.001	0.71 (0.60–0.85)	< 0.001	0.77 (0.64–0.93)	0.007
RDW						
Low level	Ref		Ref		Ref	
High level	2.05 (1.72–2.45)	< 0.001	1.34 (1.11–1.62)	0.002	1.31 (1.08–1.61)	0.007
RAR						
Low level	Ref		Ref		Ref	
High level	2.84 (2.36–3.42)	< 0.001	1.76 (1.45–2.13)	< 0.001	1.65 (1.34–2.03)	< 0.001

Model 1 was the univariate Cox model (unadjusted); Model 2: adjusted for age, vasopressor use, sequential organ failure assessment score, simplified acute physiology score II score, glasgow coma scale score and elixhauser comorbidity index score; Model 3: adjusted for age, marital status, respiratory rate, heart rate, diastolic blood pressure, white blood cell, potassium, calcium, pH, creatinine, bicarbonate, hemoglobin, pulse oximetry-derived oxygen saturation, intensive care unit length, vasopressor use, sequential organ failure assessment score, simplified acute physiology score II score, glasgow coma scale score, elixhauser comorbidity index score, percutaneous coronary intervention and coronary artery bypass graft

HR, hazard ratio; CI, confidence interval; Ref, reference; RDW, red blood cell distribution width; RAR, the ratio of the RDW to albumin; AMI: acute myocardial infarction

### Subgroup analyses based on the age, gender, SAPS II score, ECI score, treatment modalities and WBC

**Fig. 4:**
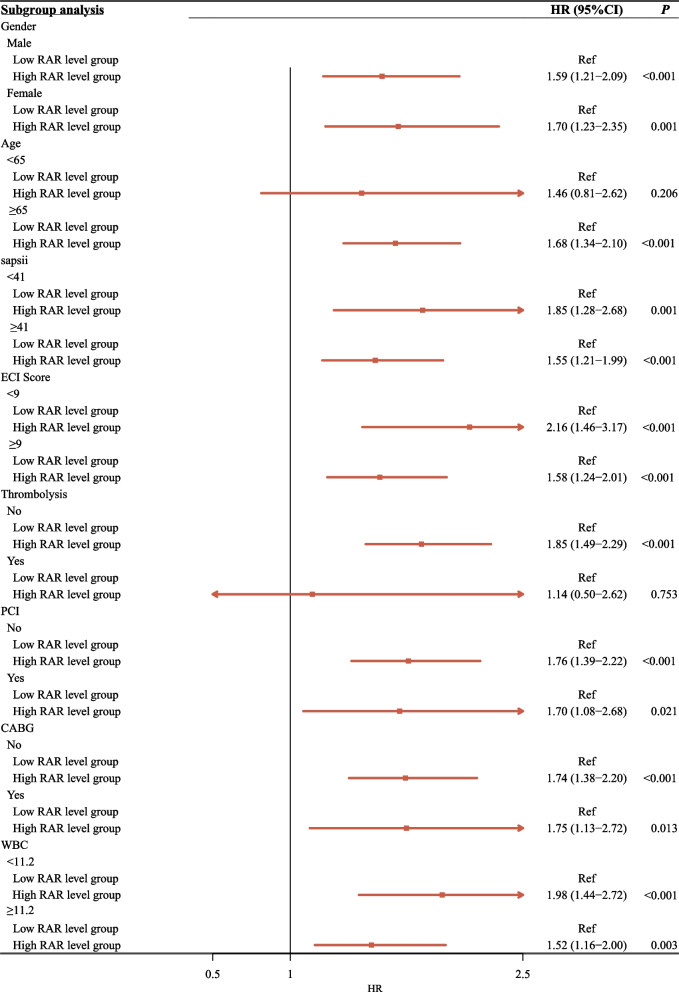
The subgroup analyses based on the age, gender, SAPS II score, ECI score, thrombolysis, PCI, CABG and WBC. SAPS, simplified acute physiology score; ECI, elixhauser comorbidity index; PCI, percutaneous coronary intervention; CABG, coronary artery bypass graft; WBC, white blood cell.

In the subgroup analyses conducted for age, gender, SAPS II score, ECI score, treatment modalities and WBC (Fig. 4), the results implied that AMI patients with the high RAR level had higher risk of 90-day mortality than those with low RAR level in subgroup analyses, except for those patients with aged < 65 years and those receiving thrombolysis.

### Predicting value

In addition, we also developed a RAR-model for predicting the 90-day mortality of AMI patients, and compared the predicting performance of RAR-model and RDW-model and albumin-model (Table 3). The C-indexes of the RAR-model were 0.610 (95% CI 0.586–0.634) in the training set, and 0.629 (95% CI 0.593–0.665) in the testing set, which were higher than RDW model and albumin model, respectively.

**Table 3:** The predicting performance of RAR-model and RDW-model and albumin-model

Models	Training set (n = 1457)		Testing set (n = 624)	
	C-index (95%CI)	*P*	C-index (95%CI)	*P*
RDW-model	0.594 (0.569–0.619)	Ref	0.592 (0.555–0.629)	Ref
Albumin-model	0.574 (0.548–0.599)	< 0.001	0.606 (0.569–0.643)	0.074
RAR-model	0.610 (0.586–0.634)	< 0.001	0.629 (0.593–0.665)	< 0.001

HR, hazard ratio; CI, confidence interval; RDW, red blood cell distribution width; RAR, the ratio of the RDW to albumin; Ref, reference

## Discussion

In the present study, we found that elevated RAR level was related to an increased risk of 90-day mortality of patients with AMI. Additionally, the finding also indicated that RAR might be a more effective biomarker in predicting 90-day mortality of patients with AMI than albumin and RDW.

RDW, commonly measured by using a blood analyzer, plays a role in the states of inflammation, poor nutrition, and microcirculation impairment [18, 19]. Previous studies have shown that RDW level were associated with the risk of mortality of AMI patients: the higher the RDW level, the higher the mortality of AMI patients [5, 20]. The mechanisms underlying the association may be as follows: some studies have pointed out that increased levels of RDW indicate reduced RBC deformability and impaired microcirculatory blood flow, which may lead to reduced oxygen supply at the tissue level, resulting in an imbalance between oxygen supply and demand that increases the risk of adverse events [21, 22]. Moreover, for patients with AMI, inflammation and oxidative stress may increase RDW level by compromising iron metabolism and shortening the lifespan of RBC, thereby modulating the response to erythropoietin through bone marrow [23, 24]. Similarly, albumin, as an inflammation biomarker, has been reported to be related to the prognosis of coronary artery disease [25] and AMI [14]. Djoussé et al. have proposed that after adjusting for age, total cholesterol, and hypertension, low albumin level was associated with an increased risk of myocardial infarction [26]. Low albumin level plays a proinflammatory role and was associated with increased oxidative stress, platelet activation and aggregation, raising all-cause mortality risk in AMI [27]. Similar to these results, our study showed that high RDW level was interconnected with an increased risk of death for AMI patients, and high albumin level might be a protective factor of death risk in patients with AMI. In the study of Li et al., they reported that RAR (RDW-to-albumin Ratio) was an independent predictor for 30-day mortality of patients with first-onset AMI [28]; however, they did not consider the effect of severity score as well as treatment modality. Remarkably, this study indicated that after adjusted for age, marital status, respiratory rate, heart rate, DBP, WBC, potassium, calcium, pH, creatinine, bicarbonate, hemoglobin, SpO2, vasopressor use, SOFA score, SAPS II score, GCS score ECI score, PCI, CABG and ICU length, high RAR level was a risk factor of the 90-day mortality for AMI patients, which may be related to the fact that both RDW and albumin were independent predictors of AMI. In addition, the subgroup analysis also displayed that the relationship of RAR level and the mortality of AMI patients was stable.

Although albumin levels and RDW level could be used as predictors of AMI death, this present study suggested that the RAR level may have a better predicting performance of 90-day mortality in AMI patients than albumin and RDW. In addition, RAR can also be obtained quickly and simply from the admission laboratory. Therefore, RAR may be a simple and readily available biomarker for prediction of death risk in AMI patients.

Nevertheless, there are also some drawbacks in this study. Firstly, since all data of participants in this study were from MIMIC-III database which is a retrospective study conducted in a single-center, the results may have a potential selection bias. Secondly, only patients with AMI in the ICU were included in our study, therefore, the relationship of RAR level and AMI patients who were not admitted to the ICU could not be determined. Thirdly, therapeutic strategies for AMI have evolved further over the decade, such as the evolution of antiplatelet drugs and drug eluting stents and the change of Dual Anti-platelet Therapy (DAPT) duration [29]. But patients’ information on the evolution of antiplatelet drugs and drug eluting stents, as well as DAPT duration was lacking in the MIMIC-III database. this may also be confounding factors in this study. Lastly, patients’ information on Killip classification [30], Thrombolysis in Myocardial Infarction (TIMI) risk score [31], peak creatine kinase (CK) values [30], left ventricular ejection fraction [31], and revascularization [32] was not collected in the MIMIC-III database, which may also be confounding factors. More prospective with multi-center studies will be conducted in the future to validate our findings.

## Conclusion

We found that RAR was a potential prognostic marker for AMI patients, and a high RAR level was correlated with increased risk of 90-day mortality for patients with AMI. In the future, RAR may be a simple and readily available biomarker for prediction of death risk in AMI patients.

## Data Availability

The datasets generated and/or analyzed during the current study are available in the MIMIC-III database, https://mimic.physionet.org/iii/.

## Abbreviations

AMI: Acute myocardial infarction
RDW: Red blood cell distribution width
RBC: Red blood cell
RAR: RDW/albumin ratio
ICU: Intensive care unit
MIMIC-III: Medical Information Mart for Intensive Care III
ICD-9: International Classification of Diseases-9
SBP: Systolic blood pressure
SpO_2_: Oximetry-derived oxygen saturation
CK-MB: Creatine kinase isoenzymes
WBC: White blood cell
BUN: Blood urea nitrogen
SOFA: Sequential organ failure assessment
SAPS II: Simplified acute physiology score II
GCS: Glasgow coma scale
ECI: Elixhauser comorbidity index
PCI: Percutaneous coronary intervention
CABG: Coronary artery bypass graft
Mean ± SD: Mean ± standard deviation
HR: Hazard ratio
CI: Confidence interval
RCS: Restricted cubic spline
